# Removal of Dyes by Polymer-Enhanced Ultrafiltration: An Overview

**DOI:** 10.3390/polym13193450

**Published:** 2021-10-08

**Authors:** Estefanía Oyarce, Karina Roa, Andrés Boulett, Sebastián Sotelo, Plinio Cantero-López, Julio Sánchez, Bernabé L. Rivas

**Affiliations:** 1 Departamento de Ciencias del Ambiente, Facultad de Química y Biología, Universidad de Santiago de Chile (USACH), Santiago 9170022, Chile; estefania.oyarce@usach.cl (E.O.); karina.roa@usach.cl (K.R.); andres.boulett@usach.cl (A.B.); sebastian.soteloa@utem.cl (S.S.); 2 Relativistic Molecular Physics Group (ReMoPh), PhD Program in Molecular Physical Chemistry, Facultad de Ciencias Exactas, Universidad Andres Bello, República 275, Santiago 8370143, Chile; pliniocantero@gmail.com; 3 Center of Applied Nanoscience (CANS), Facultad de Ciencias Exactas, Universidad Andres Bello, Av. República 330, Santiago 8370186, Chile; 4 Polymer Department, Faculty of Chemistry, University of Concepción, Concepción 4030000, Chile; brivas@udec.cl

**Keywords:** dyes, membranes, ultrafiltration, washing method, water-soluble polymer

## Abstract

The current problem of contamination caused by colored industrial effluents has led to the development of different techniques to remove these species from water. One of them, polymer-enhanced ultrafiltration (PEUF), has been systematically studied in this mini review, in which research works from 1971 to date were found and analyzed. Dye retention rates of up to 99% were obtained in several cases. In addition, a brief discussion of different parameters, such as pH, interfering salts, type of polymer, dye concentration, and membrane type, and their influence in dye removal is presented. It was concluded from the above that these factors can be adapted depending on the pollutant to be remediated, in order to optimize the process. Finally, theoretical approaches have been used to understand the intermolecular interactions, and development of the studied technique. In this revision, it is possible to observe that molecular docking, molecular dynamics simulations, density functional theory calculations, and hybrid neural-genetic algorithms based on an evolutionary approach are the most usual approximations used for this purpose. Herein, there is a detailed discussion about what was carried out in order to contribute to the research development of this important science field.

## 1. Introduction

Water pollution is a big concern around the world [1]. The problem in industries such as textiles, paper, plastics, leather, food processing and cosmetics is the waste they generate due to their manufacturing processes: they produce large quantities of organic and inorganic pollutants, such as sludge, acids, bases, phenols, etc. [2]. Among them, dyes are considered one of the most dangerous contaminants because they have very recalcitrant structures [3]. In addition, they represent a serious threat to global environmental sustainability since, in the aquatic environment, they hinder the penetration of light, causing flora not to carry out its photosynthesis process [4,5]. Moreover, the discharge of poorly fixed dyes into the terrestrial environment, together with industrial waste, causes an imbalance of nutrients affecting soil toxicology in agricultural fields, preventing the germination of seeds and the development of plants [6]. Dyes are also a threat to human health, because the intake of these dyes at small concentrations can cause cancer, allergy, dermatitis, and mutagenesis, among other health issues [2,6,7]. Accordingly, several techniques have been developed to solve this problem. Some of these techniques are coagulation/flocculation [8], biological treatment [9], advanced oxidation process [10], adsorption [11,12], and membrane processing [13,14]. Among membrane processes, pressurized liquid filtration techniques have become an essential treatment in industries that generate large quantities of wastewater [15]. These membrane techniques are reverse osmosis (RO) [16], nanofiltration (NF) [16], microfiltration, (MF) [17], and ultrafiltration (UF) [18]. However, reverse osmosis (RO) and nanofiltration (NF) processes have a low permeability, thus they require high pressure for operation, which increases the cost of processing [19,20]. On the other hand, the microfiltration (MF) and ultrafiltration (UF) processes have a high permeability, so the operating pressure is low compared to those of the other techniques mentioned; however, they are ineffective for the retention of small molecules with a low molecular weight such as dyes [21,22,23,24,25]. Therefore, the use of ultrafiltration (UF) has an advantage over other types of filtration since it reduces the energy cost [26,27] and water-soluble surfactants or water-soluble polymers can be added to improve the retention of dyes [21]. The UF combined with water-soluble surfactants is called micellar-enhanced ultrafiltration (MEUF), and the UF combined with water-soluble polymers is called polymer-enhanced ultrafiltration (PEUF). In PEUF, the binding of macromolecular ligands has a high capacity compared to MEUF, which is expected to provide sufficient permeate flow with high efficiency [28,29]. In addition, from an intermolecular point of view, the macromolecular complexation cooperates to produce better adsorption capability with respect to the use of the UF technique alone. Polymer–dye interactions are thermodynamically favorable, normally due to the presence of electrostatic, hydrophobic interactions, among others, which increase the solute retention performance [6,30,31]. Further advantages are that PEUF is environmentally friendly, is a continuous method, and easy to adapt to the characteristics of the effluent and the nature of the pollutant [6]. However, the operating principle of conventional ultrafiltration is the same as that of PEUF. The unique difference is the addition of a water-soluble polymer, which is added into the feed solution to capture the corresponding dye [16].

In this work, the term PEUF will be used for convenience and the topic will focus on PEUF for the removal of dyes in aqueous solution. A critical review of the current state of the art will be presented, emphasizing the removal of methylene blue (MB) and methyl orange (MO) dyes, both widely used in industry. Additionally, the different dyes in wastewater and the operation of the PEUF technique with different water-soluble polymers will be discussed. Considering the chemical nature of polymers, as well as their interaction with dyes as a function of different experimental variables (pH, concentration of dye, soluble polymer concentration, the effect of ionic strength, and the type and molecular cut-off size of the UF membrane), the removal of dyes from aqueous solution is evaluated. In this context, during recent decades the polymer assisted ultrafiltration (PEUF) technique has received great attention from the scientific community because it has proven to be efficient in the removal of organic contaminants from wastewaters [21,31,32,33]. Despite the importance of this topic, to date few research works about the uses of this technique have been reported [32,34,35]. Therefore, this review is focused on the discussion of this technique, as well as the recent advances, and perspectives for future research related to the removal of dyes through the PEUF technique, taking into account experimental and theoretical works.

## 2. Dyes in Industrial Wastewater

Dyes are defined as substances that when applied to a substrate provide changes in the crystalline structure, thus changing the interaction with light by reflecting a different visible wavelength that consequently reflects the color of the dye [32,36]. Responsible for providing color to the dyes is a group of atoms called chromophores (electron acceptors) which are represented by the following radicals: azo (-N=N-), carbonyl (=C=O), carbon (=C=C=C=), carbon nitrogen (=C=NH or -CH=N-), nitrous (-NO or N-OH), nitric (-NO_2_ or =NO-OH), and sulfur (=C=S) [7,37,38,39]. Responsible for dyeing ability is a group of atoms called auxochromes (electron donors) which are usually aromatic structures containing benzene, naphthalene or anthracene rings. These are ionizable, responsible for the binding ability of the dye molecules on the substrate [40]. Common auxochrome groups are: -COOH (carboxyl), -NH_2_ (amino), -SO_3_H (sulfonic acid), and -OH (hydroxyl) [41]. At the industrial level the most frequently used dyes are those with azo, anthraquinone, sulfur, indigoid, triphenylmethyl, and stacyanine in their chemical structure [42]. There are natural colorants that are extracted from a vegetable, animal, or mineral substance, such as curcumin (E100), carminic acid (E120), xanthophylls (E161), charcoal (E153), etc. The problem with these natural dyes is their limited existing variety and their opaque hues that fade by the action of light and washing [43]. Therefore, the need arose to modify or create new dyes in order to improve their properties and to create new color shades [37,44]. This gave rise to the production of synthetic colorants that are synthesized in laboratories, mostly from oil [42]. Studies reveal that in every year more than 100,000 tons of dyes are synthesized in the world and 10% of this tonnage is discharged into the environment, causing serious problems to human health and aquatic organisms [45]. Some of these dyes possess nitrogen atoms in their molecular structure, being very harmful to the aquatic life cycle, plants, and animals [46]. In addition, they hinder the penetration of sunlight into the waters, seriously impairing the performance of photosynthesis in microorganisms responsible for the production of oxygen in the aqueous medium [41].

**Figure 1:**
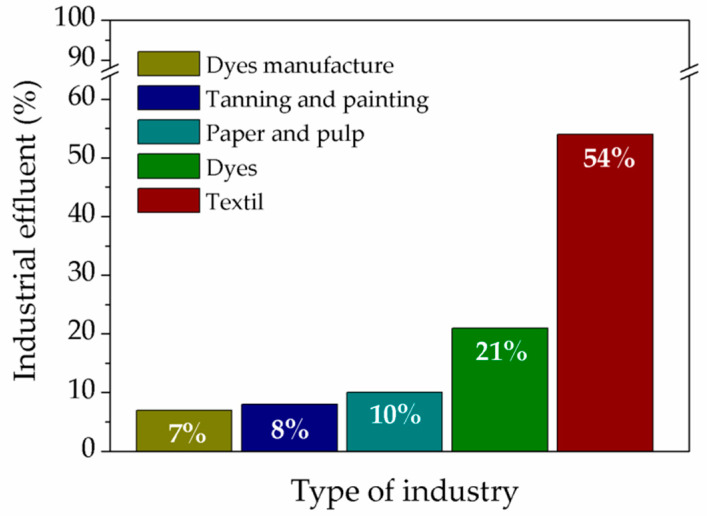
Dye-contaminated wastewater generating industries.

It is known that there are five major industrial sectors that generate wastewater that contain dyes and are responsible for the presence of dyes in the environment [47]. This is shown in Figure 1, where it is possible to see that 54% of the released effluents correspond to the textile industry, 21% to the dye industry, 10% to the paper and pulp industry, 8% to tanning and painting industries, and 7% corresponds to the paint and pigment manufacturing industry [43].

The most commonly used classification to differentiate the types of synthetic dyes with respect to their chemical nature after dissolving in water is: anionic (including acid dyes, reactive dyes, azo), cationic dyes (include basic dyes), and non-ionic dyes (including dispersing dyes that do not ionize in aqueous media) [42]. However, ionic dyes are the main objects of studies investigating removal by PEUF. One of them is methylene blue (MB) since it is the most widely used cationic dye at an industrial level in the wood, paper, silk, leather, plastic, cosmetic, pharmaceutical, and food industries [46]. This organic dye is dark green in color, but in aqueous solution it is blue. It has several uses, such as staining cells or bacteria for microscopic observation, as an antiseptic and disinfectant, and is also used as a reagent for chemical analysis [48,49,50,51]. It also has redox indicator properties, since it is blue in an oxidizing environment and loses its color in a reducing environment [52]. This dye can cause eye burns as well as respiratory problems when inhaled, while when ingested through the mouth it causes burning, nausea, vomiting, deep sweating, mental confusion, and methemoglobinemia [53].

Another dye widely used at the industrial level is methyl orange (MO). Industries such as pharmaceuticals and textiles use this dye; it is also used as an acid-base indicator. It is an azoderivative chemical compound with weak acid characteristics, which at a pH between 3.1 and 4.4 changes from red to orange-yellow [54]. The MO molecule has a bright orange color when dissolved in water, a stable chemical structure due to the presence of azo, and aromatic groups which are highly toxic, carcinogenic, and teratogenic, and are harmful to the environment and organisms because it shows low biodegradability [55].

## 3. Polymer-Enhanced Ultrafiltration (PEUF) Technique

The PEUF technique consists of the addition of a high molecular weight water-soluble polymer, with the objective of separating low molecular weight species such as heavy metals, dyes, dissolved organic compounds, etc. [15,56,57]. The technique is based on the fact that the polymer interacts with the dissolved solute present in wastewater, increasing in size, and being able to be retained by a membrane that can separate molecules of sizes in the order of 100 nm [15]. This description not only corresponds to PEUF, but also to complexation-ultrafiltration, polymer-supported ultrafiltration, polyelectrolyte-assisted/enhanced ultrafiltration and polymer-assisted/PEUF, which basically refers to the same technique of separation of small pollutant molecules from water [58,59]. In 1968, Michaels et al. were the pioneers in publishing this technique, which developed the hybrid complexation-ultrafiltration process to selectively separate small molecules of dyes and metal ions from aqueous solutions [60]. Later, Izumi et al. determined thermodynamic parameters to study the temperature dependence of dye–polymer interaction in aqueous solution [61]. The binding behavior between the dye and the polymer depends entirely on the chemical structure of the polymer and dye, so it cannot be generalized [15]. The polymer–dye interaction is hydrophobic and energetic, and involves electrostatic interactions, chemical interactions, and hydrogen bonding [15].

**Figure 2:**
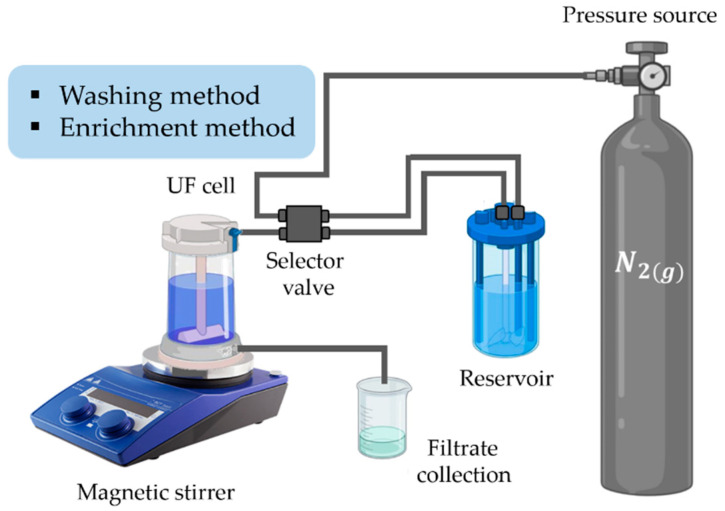
Schematization of the PEUF system.

The system of the PEUF technique is mainly constituted by: a membrane, a stirred cell filtration unit, a reservoir or storage tank, a manometer, and a pressure pump with nitrogen gas (N_2_) (see Figure 2) [30,59,62]. The membrane is an essential component for the PEUF technique, and must possess good chemical resistance, thermal stability, high mechanical strength, and the ability to form tubular or flat membranes, as well as surface aspects such as surface wettability, surface charge, and surface roughness [63]. The membrane can be also modified in order to improve its performance (mixing, grafting, and the use of suitable additives such as pore formers) [64,65,66,67].

There are two types of separation by PEUF, the washing method and the enrichment method [68,69]. The washing method consists of eluting an aqueous solution, at a certain pH and ionic strength, to a polymer–dyes solution (of known concentration of dye containing a certain amount of polymer) [68]. In contrast, the enrichment method consists of eluting a solution containing a certain amount of polymer with a solution of known concentration of dye [68]. The polymer begins to come into contact with the colorant, increasing the concentration of the colorant in the UF cell [70]. In both cases, the UF cell solution and the eluent solution have the same pH value. The processes are carried out in a certain time, during which the pressure in the system remains constant [71].

The setting of parameters in the PEUF system, such as pH, dye, and polymer concentration, will depend exclusively on the chemical structure of the polymer and dye to be removed from the contaminated effluent [21,31,32,33,38]. For example, Mondal et al. used two polyelectrolytes, poly(acrylic acid) (PAA) and poly(ammonium chloride acrylate) (PANH_4_), in the PEUF technique for the removal of three different dyes: crystal violet (CV), eriochrome blue-black R (EBBR), and saffarin T (ST) [72]. They used a regenerated cellulose membrane with a molecular weight cut-off (MWCO) of 10 kDa, at a fixed transmembrane pressure of 2 bar at room temperature (25 °C). The pH range for CV was 6–10, for EBBR 0–10, and for ST 7–12 [72]. The concentration for all dyes in this study was 10 mg L^−1^ and sodium chloride was used as an interferent [28]. In another study, Dasgupta et al. applied a strategy based on a stochastic genetic algorithm coupled with an artificial neural network to optimize the retention of reactive red dye 120 (RR 120) [73]. For this purpose, polyethylenimine (PEI) with a 30 kDa polyethersulfone (PES) membrane was used, achieving in an optimal configuration: a rejection rate of 99.9% at pH 5.7 and an initial concentration of 50 mg L^−1^ of dye [73].

### 3.1. MB Removal by PEUF

Some investigations have been reported regarding the elimination of MB dye through PEUF, where the interaction capacity of this dye with different water-soluble polymers and the membrane is analyzed [28,31,33,74,75,76]. In one of the investigations reported in the literature, Moreno-Villoslada et al. studied by UV–vis the type of interaction of MB dye with different soluble ionic polymers [76]. The interactions can be of the long-range electrostatic, hydrophobic, or short-range type (ar/ar interactions) between MB (cationic dye) and three different water-soluble polymers selected because they possess in their chemical structure the sulfonate group (SO_3_^−^): poly(sodium 4-styrene sulfonate) (PSS), poly(sodium vinylsulfonate) (PVS), and poly[sodium 2-(N-acrylamido)-2-methylpropanesulfonate] (PAMPS) [76]. It was determined that in the case of PSS, a polymer with an aromatic ring, the predominant interaction is of the short-range ar/ar type, although there is also a complementation with short-range electrostatic forces and nonspecific hydrophobic interactions [76]. In addition, when NaCl is added, the interactions are only affected at low polymer concentrations. In the presence of an excess PSS, the interactions are preserved without variation [76]. In the case of PVS, the main interactions are due to long-range forces [76]. PAMPS exhibits a higher hydrophobic character due to dangling aliphatic chains, so it is expected that the interactions with MB result from the contribution of hydrophobic forces. Using UV–vis, a similar behavior to that of PVS was observed, with a lower formation of polymer–MB aggregates [76]. By adding NaCl, the interaction with the dye is also quenched. It can be summarized that the effect of the interferent in the polymer–dye interaction decreases in the order PSS > PAMPS > PVS [76].

On the other hand, Oyarce et al. evaluated the removal of MB using a sodium alginate (SA) biopolymer, evaluating the effectiveness by varying the pH, SA dose, MB concentration, and SA reuse, obtaining a retention capacity of 98% of the dye at pH 8 using 0.025 g of SA, at an initial concentration of 50 mg L^−1^ [28]. In the reuse study, it was seen to work in five consecutive dye adsorption–desorption cycles. The MB retention efficiency in the last cycle was 87%, demonstrating the capacity of polymer regeneration and its posterior reuse in the PEUF process [28].

### 3.2. MO Removal by PEUF

Few investigations have been reported regarding the removal of MO dye through PEUF [2,18,77,78]. Fradj et al. studied the removal of MO by PEUF, using chitosan (CHI) with a molecular weight of 117 kDa, obtaining a retention rate of 86% at pH 4.5 at 25 °C with a polymer dose of 0.4 mM and an initial MO concentration of 0.1 mM (32 mg L^−1^) [2]. In this study, it was concluded that the efficiency of the process was due to the formation of a complex between the azo dye and the cationic polyelectrolyte [2].

In addition, apart from MB and MO, PEUF has been used in the removal of several ionic dyes such as: direct blue 71 (DB71), EBBR, RR 120, acid-orange 7 (AO7), carmine indigo (IC), amide black (AB), direct black (DB), reactive black 5 (RB5), acid blue 129 (AB129), orange II (OII), ethyl orange (EO), ST, malachite green (MG), bright green (BG), new fuchsia (NF), CV, MB, toluidine blue (TB), and acridine yellow (AY) [2,6,18,21,31,60,63,73,76,77,78,79,80,81,82,83,84,85]. Table 1 shows the classification of the dyes according to their chemical speciation in solution and their molecular structure.

Through a systematic study of the literature, 24 scientific reports related to the removal of dyes by PEUF were found between 1986 and the year 2021, revealing the few publications on this subject. It should be noted that the dye most studied so far is MB [28,31,33,74,75,76], followed by MO [2,18,77,78,79].

In addition, it was found that the water-soluble polymers most used for dye removal studies by PEUF are PAA [21,31,72,80] and PEI [73,74,81,82]. Both polymers contain an acid and amine as the active functional moiety, respectively. The regenerated cellulose UF membrane is the most used in the PEUF process [2,18,21,28,31,70,72,76,77,78,79,82].

## 4. Synthetic and Natural Water-Soluble Polymers Used as Extractants of Dyes

Water-soluble polymers (WSPs) are the most important component in PEUF [15,87]. WSP interacts with dye molecules in solution, through attractive forces, resulting in the formation of large agglomerates or macromolecular complexes of WSP–dyes [2,15,85]. This allows the dye to be retained in the UF cell and it does not elute through the membrane to the permeate.

**Figure 3:**
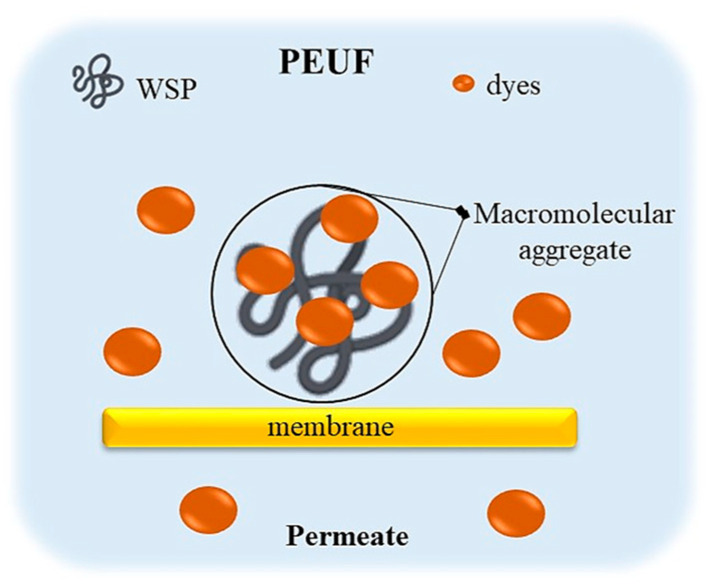
Schematic representation of the formation of macromolecular aggregates of WSP–dyes in the PEUF process.

Consequently, a permeate with little or no presence of colorants is obtained [80] (see Figure 3). In general, water-soluble polymers can be classified into two categories: polyelectrolytes (polymers with charged groups in their repeating units) and polychelatogens (chelating polymers that contain chelating groups or complexes) [15,68,88,89,90,91,92,93,94]. However, the most commonly used WSP for the removal of dyes in aqueous solution are polyelectrolytes. These contain groups easily ionizable in water, such as -OH, -COOH, -NH_2_, -HSO_2_ [28,68,88,89,90,91,92,93,94], which are able to obtain polymers in solution with a positive or negative charge, called anionic or cationic polymers. The high content of functional groups contributes to the extractant properties of these compounds [28,95,96]. The ionic nature of polyelectrolytes in solution generally induces electrostatic or ion exchange attractions between adsorbate–adsorbent [15,68,88,89,90,91,92,93,94]. The use of easily ionizable WSPs facilitates the uptake of acidic and basic dyes in the PEUF process, where acid dyes are attracted to cationic polymers and basic dyes to anionic polymers, respectively [2,72,75].

Dasgupta et al. used the synthetic polymer PEI and the CHI biopolymer for their studies of removal of RR 120 from aqueous solutions via PEUF [6]. Both WSPs are cationic in nature, attracting anionic dyes through the amino groups present in their molecular structure [6]. The researchers carefully studied the formation of dye–polyelectrolyte complexes. After incorporation of a polyelectrolyte into the solution, they reported UV–vis spectrophotometry results on the characteristic changes in the maximum observable wavelength of RR 120 (λ_maximum_: 506 nm) [6]. They indicated that the change in λ_maximum_ is essentially attributed to the transition of the dye molecules from the free or unconsolidated state to the polymer-bound state [6], as well as concomitant conformational changes in the dye molecules and the polymer. The aggregation of the molecules, and the formation of macromolecular aggregates of WSP–dyes, reduces the total energy of the system by reducing the degree of contact between the hydrophobic residues of the dye ions and the water, and improves the entropy of the system. The maximum bathochromic shift of λ_maximum_ for the CHI-RR 120 systems was relatively insignificant. It is a consequence of the stereochemistry of the CHI [6]. The binding constant of the PEI-RR 120 and CHI-RR 120 complexes indicated that PEI is the most suitable complex agent for the removal of RR 120, with these WSPs reaching a removal efficiency of RR 120 of 99.9% and 88%, respectively [6].

There are also studies on the binding of dyes to different synthetic and natural polymers by means of absorbance, fluorescence, and determination of thermodynamic parameters, which during recent decades produced significant contributions to advances in PEUF technology [33,61,74,84]. Moreno-Villoslada et al. studied the interaction of MB with PSS, PVS, and the more hydrophobic PAMPS using UV [76]. For PVS, a decrease of the monomer band was observed in an equimolar way to the concentration of MB and the appearance of a band at 570 nm was observed, which is called poly-MB [76]. The appearance of this band was justified in this study, which stated that it was due to the formation of aggregates by the accumulation of a high concentration of dyes in the surroundings of the polyanions due to the action of long-range forces and the tendency of MB to self-aggregate [76]. In the case of PAMPS, this polymer shows similar behavior to PVS but a tendency to avoid MB aggregates is observed. PSS is a polymer containing a single aromatic ring to the sulfonate group, which presents the ability to bind aromatic cations by short-range electrostatic forces, ar/ar type interactions, and hydrophobic interactions [76]. In the UV spectrum, a band shift towards 616 nm was observed, which shows the formation of small aggregates, as well as an increase in the monomer band with increasing polymer concentration, indicating a high dispersion of the dye [76]. On the other hand, the same study corroborated by spectroscopy H-NMR the ar/ar type interaction between PSS and MB, and that it predominates the self-aggregation of MB [76].

The solubility of WSPs in aqueous matrices is also one of the important general requirements to consider [15]. This is so since if the interaction between polymeric molecules and water is less than the interaction between the molecules in each component (water–water and/or polymer–polymer), the same molecules will tend to join or agglomerate, forming two phases [15]. The latter does not favor the proper functioning of the PEUF process [15]. However, as previously mentioned, the WSPs used to capture dyes are mostly polyelectrolytes that have permanent dipole moments. Polymeric polar molecules when in contact with water acquire a high affinity for it [15]. Where the dipole–dipole interactions between the polymer and water molecules are effective and, consequently, generate a greater interaction compared to the interaction between water–water molecules and/or polymer–polymer, so the polymer is solubilized in water [15]. Another requirement to consider is the molecular weight of the WSP, which must be higher than the MWCO of the UF membranes used in the process [15,28,88]. A molecular weight higher than the MWCO of the UF membrane favors the non-passage of WSP through it, avoiding its elution to the permeate [15]. This makes the WSP stay inside the UF cell, allowing a correct operation of the dye removal process. In this context, synthetic polymers and biopolymers have been used for the removal of dyes by PEUF, synthetic polymers such as PAA, PSS, PEI, and poly (ammonium acrylate) (PANH_4_) being the most studied, as shown in Table 2.

## 5. Removal of Dyes on Function of Different Variables

### 5.1. Effect of pH on Dye Removal

**Figure 4:**
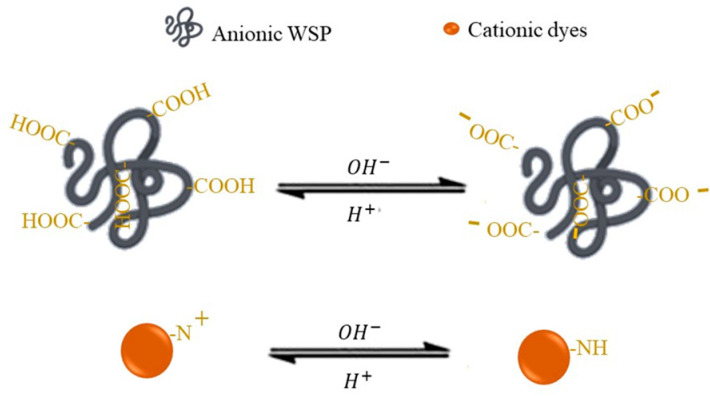
Schematic representation of the effect of the pH of the solution on the functional groups of both the polymer and the dye.

The pH of the solution is an important parameter in the PEUF process as it can disturb the retention rate and the degree of ionization of the soluble polymer and dye (see Figure 4). Thus, to study it, the other variables are fixed by varying the pH in a certain range. In the case of Fradj et. al, they varied the pH by adding hydrochloric acid and sodium hydroxide, observing an optimum pH of 6, which is justified by the presence of cationic amino group (-NH_3_^+^) due to the protonation of CHI (pK_a_ = 6.3) [97]. Another important factor is the pK_a_ of the dye, which in the case of this study is MO (pK_a_ = 3.42) [2]. So in this research the pH optimum of 6 can be explained by the electrostatic interaction and complex formation between the sulfonate groups of the dye and the cations formed by the polyelectrolyte [2]. A study by Ben Fradj et al. found an optimum pH for MB and TB removal in the basic range (5–14) for both polyelectrolytes used [33]. This is due to higher acidic pHs preventing the dissociation of the dye and the carboxylic group of the PAA [33]. In the case of PANH_4_ it reaches its optimum value at 8 because at that pH ammonium hydroxide is formed in the solution, which causes an increase in the dissociation of the polymer [21]. A similar situation was studied in 2014 by the same author where he justifies it by the PAA pK_a_ of 4.28, which leads to a lower retention at pH lower than this value; the same interpretation is given for PANH_4_ with its pK_a_ = 5.8, because the interaction is mainly of the electrostatic type [31]. In the case of Dasgupta et. al, the retention of an acid dye RR 120 was studied with CHI and PEI as polyelectrolytes. In both cases there was a low retention rate at pH close to the pK_a_ of the polymers studied; 8.4 for the case of PEI and 6.3 for CHI [98]. The drop in dye retention at pH > 9 is mainly due to base-acid interactions between the dye and the amino group. The difference in retention rates, close to full in the case of PEI and close to 90% in the case of CHI, may be due to the higher amount of amino groups present in the former, compared to only the primary amino groups in the case of CHI [6]. On the other hand, Ouni et al. studied the effect of a pH in a range of 2–14 for the removal of CV, observing a retention of 99% at a pH above 4. In the case of PEI, a maximum retention was obtained at a pH above 10 [86], which coincides with the work of Li et. al, which can be explained by the pK_a_ of PAA [99]. Ouni et al. studied how the use of a polymer as a surfactant affects the removal of MO using PEUF. In the study a pH range of 2–12 was used, achieving a maximum removal of 70% without the presence of the surfactant octadecyltrimethylammonium bromide (CTAB), remaining constant above this pH, which can be explained by the pK_a_ = 3.7 of the MO, which also presents a color change of the dye [78]. MO is quinoid in acidic media which is more stable than its azo form, so in its acid form it blocks retention [78]. On the other hand, when CTAB (0.4 mM) was applied, there was no longer a change in the retention of MO at a different pH, and a retention of 99% was obtained throughout the range studied [78].

### 5.2. Effect of Ionic Strength on Dye Removal

The effect of ionic forces is studied by modifying pH or adding interfering salts [100], observing the modification of the absorption spectrum by UV–vis spectrophotometry [86]. Fradj et al. studied the removal rate of MB and TB using PAA, varying the pH in the range 2–11, where it was known that the pK_a_ of the polymer was 4.28 [99]; therefore, at a lower pH than this there was no interaction with the dyes. At a higher pH it was observed by UV–vis spectroscopy that there was complex formation between the dyes and polyelectrolytes, mainly in the anionic form. At pH = 10.86, a decrease in electrostatic forces was observed; this was verified by the increase in absorbance of the monomer form [33]. Ouni et al. meanwhile studied the effect of ionic forces in CV removal using PAA and PEI as polyelectrolytes [86]. They demonstrated how it affects the addition of NaCl in a range of 0.001–0.5 M, where for PAA a clear decrease in absorbance was observed [86]. A displacement of the maximum from 565 to 541 nm indicated a disintegration of the dye, which can also be attributed to the presence of Na^+^ cations that competed with the dye to bind to the charged sites of the polyelectrolyte, which decreased the formation of dye-polyelectrolyte complexes [86]. For PEI, there was a shift from 604 to 592 upon addition of the minimum amount of salt, then upon further incorporation in the study range, there was no more variation [86], a situation similar to that observed by Ouyang et al. [101]. Tan et al. studied the UV–vis absorption spectrum of BG and MG solutions in the presence of PSS where the dye concentration varied, keeping the polymer concentration fixed [85]. Under these experimental conditions, it was corroborated in the case of BG that they moved from the usual maximum of 634 to 625 nm, demonstrating the formation of complexes; in the same way, MG decreased the absorption peak from 629 to 617 nm [85].

### 5.3. Effect of Interfering Ions on Dye Removal

**Figure 5:**
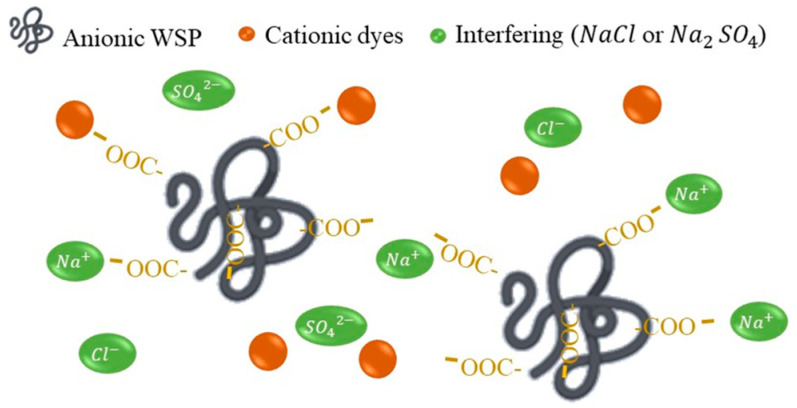
Schematic representation of the effect of interferents on the PEUF process.

In UF adsorption research, the effect of interfering salts is studied, since liquid industrial wastes usually have other components that must be studied to see how they can affect the removal efficiency. In general in investigations, NaCl is used as a model salt, where the concentration is varied in a certain range, fixing the concentration of dye and polymer [102]. Fradj et al. studied the effect of NaCl in a concentration range of 0–1500 mM, determining that the dye retention rate decreased with an increasing NaCl concentration up to 400 mM [2]. Then, above 400 mM, the percentage remained constant. This result indicates that the added salt decreases the hydrophobic and electrostatic interactions between the cationic and dye sites of the polyelectrolyte and reduces the complex formation constant [2]. The lower dye retention rate may be due to the contention of anions to bind to the positively charged sites in the polyelectrolyte; after 400 mM one can speak of an equilibrium between the two negatively charged species [103]. In the study of Ben Fradj et al. the effect of NaCl on TB retention was studied, where the electrolytes were PAA and PANH_4_ [21]. A decrease in the retention rate of the dye was observed, due to the decrease in electrostatic forces between the dye and the polyelectrolytes, reaching an equilibrium at 0.1 mol L^−1^ NaCl [21]. A similar situation was observed in the work of Fradj et. al, where the same polymers were used in the removal of MB in the presence of PAA and PANH_4_, where for both polyelectrolytes there was a decrease until equilibrium was reached at 1000 mM with a retention of 18%. This was explained by the decrease in electrostatic interactions [31]. Ouni et al. meanwhile studied the effect of NaCl on the retention rate of CV in the presence of PAA and PEI. For PAA, the dye removal decreased by 30% when adding salt from 0.0001 to 0.5 mol L^−1^ [80]. This can be explained by the lower complex formation due to the increase in ionic strength, confirming that electrostatic interactions are the main ones involved in complex formation [80,104]. In the case of PEI, there was no large decrease in the dye retention rate, confirming that complex formation is not mainly of the electrostatic type, and that permeate flux is independent of ionic strength [86]. Subsequently, Ouni et al. compared the effect of two salts, sodium sulfate (Na_2_SO_4_) and NaCl, in the removal of MO in the presence of polyethylene glycol (PEG) and CTAB [78]. In the case of MO + PEG + Na_2_SO_4,_ the presence of salt caused a decrease of 20%, due to competitiveness between similar charges [105]. By adding CTAB, which acts as a surfactant, and studying the retention of MO + PEG+ Na_2_SO_4_ + CTAB, the retention did not change, so the addition of this compound caused the independence of the ionic strength; in the case of NaCl, it caused the same effect [78]. Figure 5 shows a schematic view of the effect of interfering salts on the interaction between the soluble polymer and the dye. It is reported in the literature that NaCl and Na_2_SO_4_ are the salts generally used as interferents in the PEUF method for the removal of dyes [2,78,80,86].

### 5.4. Effect of Polymer and Dye Concentration on Dye Removal

The study of the effect of the concentration of polyelectrolytes on the removal of dyes is always evaluated in solutions with a fixed concentration of dye [28,80]. In most cases the removal efficiency of the dye increases as a function of the increase in the polymer concentration in the working solution [15]. This observation can be explained by the addition of active binding sites through WSPs within the UF cell, which causes a greater amount of dye molecules to be complexed by polymeric macromolecules [15,82,85]. However, the hydrodynamic flux has an inversely proportional relationship to the WSP concentration present in the working solution, decreasing with increasing polymer concentration in the ultrafiltration cell [76]. This effect is attributed to fouling of the UF membrane, where excess polymer manages to form a polymer or dye–polymer layer on the surface of the membrane, which generates an increase in resistance against the flow of permeate through the membrane [15,77]. The hydrodynamic flow is of importance in the process, since it is the driving force which allows part of the mobility of the polymer and dye molecules in solution, facilitating the attraction between them and allowing UF to be generated [76]. Fradj et al. observed that by increasing the CHI concentration in the UF cell, the retention rate of DB71 and MO dyes increased [2], where the complex formed WSP–dyes induced the shift from equilibrium to the formation of the complex, the polymer, and the dye [2]. The researchers inferred that each cationic site of the polycation associated with an anionic site of the corresponding dye and this induced a stacking of the dye molecules in the polymer matrix [2]. Consequently, due to the effect of the polymer concentration on the surface of the membrane and its polarization, it caused a decrease in hydrodynamic flux [2]. Fradj, in previous studies on the removal of MB by PEUF, observed that the removal efficiency increased together with the added concentration of PAA and PANH_4_ in the solution [31], inferring that, when equilibrium of complexity is reached, excess polymer has no effect on retention efficiency [31]. In summary, increasing the concentration of cationic or anionic polyelectrolytes enhances dye removal efficiency but it disfavors the hydrodynamic flow of the process [31]. For this reason, studies must be carried out, depending on the amount of polymer, to determine the appropriate concentration of WSP for the correct operation of the PEUF method.

With respect to the initial dye dose, Oyarce et al. studied the removal of MB in a range of 1 to 100 mg L^−1^ at pH 8 and a SA dose of 0.025g [28]. They determined for concentrations range 5–100 mg L^−1^ that the retention remained constant at 98% [28]. This result is attributed to the high solubility of MB in SA; therefore, based on the literature, the concentration of 50 mg L^−1^ was chosen as optimal in this study [28]. With respect to the variation of the hydrodynamic flux when varying the initial concentration, in the study of Oyarce et al. a significant decrease was not observed, which was attributed to the self-aggregation of MB previously reported by Moreno-Villoslada et al. [76] and to the deposited layer of SA-MB aggregates on the UF membrane, which caused an obstruction in the flow path and allowed a favored retention of MB [28].

### 5.5. Effect of the Membrane on Dye Removal

**Figure 6:**
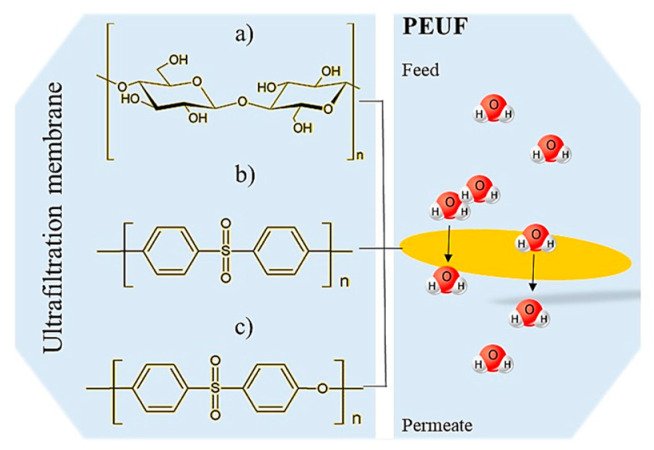
Molecular structures of components that make up UF membranes: (**a**) cellulose; (**b**) PSF; and (**c**) PES.

Through a systematic study of the literature, we found that the membranes used for PEUF are generally of cellulose, polysulfone (PSF), and PES [73,77,79,83,85]. These compounds have a high hydrophilicity, which allows the membranes to be water-permeable, as shown in Figure 6. Membranes made of regenerated cellulose are characterized by having a higher hydrophilicity [106] than PSF and PES membranes [21,77]. The easy passage of water allows a constant hydrodynamic flux in the PEUF process [15,68,94]. The pore size of the UF membrane is very important since it regulates the passage of the molecules to be eliminated by size exclusion [2,28]. The MWCO allows us to identify the associated pore size of the UF membrane [64]. PSAs with high molecular weights cannot cross membranes of approximately 5 kDa and 10 kDa [21,77]. The smaller the pore size, the greater the removal of the dye [32]. This allows the supramolecular aggregates of WSP dyes to be retained on the surface of the membrane and not eluted [15]. Therefore, the water is purified by size exclusion of the molecules present in the working solution [65,107,108]. Majewska-Nowak et al. observed that as the MWCO of the ultrafiltration membrane used in the UF process increases, the permeability of the membrane decreases [77]. They perceived a dramatic drop in the permeability of the membrane, independent of the material that constitutes it (regenerated cellulose and PES) at MWCO values over 10 kDa. Large MWCO favor the elution and/or adsorption of dye molecules on the pore walls or on the membrane surface [77].

The weak interaction that WSPs and dye have with the UF membrane prevents a fouling effect [30]. However, in practice this is not fulfilled, since minimal interactions between WSP molecules and dyes with the UF membrane can be generated [15]. In a previous investigation, Oyarce et al. studied the effect of the regenerated cellulose ultrafiltration membrane on the PEUF process for the removal of MB [28]. They evaluated the ultrafiltration of the dye in the presence and absence of WSPs [28]. They observed a 13% removal of MB without WSPs in the working solution [28]. It was attributed to an adsorption phenomenon that occurred on the surface or in the pores of the membrane [28]. Majewska-Nowak et al. observed that the excess of polymer, together with the accumulation of aggregates or macromolecular complexes on the ultrafiltration membrane, induced its polarization [77]. The hydrophilic/hydrophobic properties of the experimental membranes used and the retained substances support the above [77], where the decrease in the permeability of the ultrafiltration membrane contributes to the fouling by adsorption [77]. This is since, in the performance of the ultrafiltration membrane, the interactions of van der Waals, hydrogen bonds, electrostatic effects, charge transfer effects, and dipole moments between membrane, dye, and/or WSP play a critical role [68,88,89,90,91,92,93,94]. The fouling of the membrane is translated in the precipitous fall of the hydrodynamic flow, where hydrophilic membranes are less susceptible to irreversible adsorbate adsorption on their surface than hydrophobic membranes [99]. Therefore, the permeability of the solvents and the retention of solutes are highly correlated with the structures and sizes of the pores of the ultrafiltration membrane, together with the materials that compose it [15,68]. However, the causes that generate fouling are variable, including a reversible reduction in flux, as the result of concentration polarization and an irreversible reduction, such as fouling of the ultrafiltration membrane [109,110]. The study of different experimental parameters contributes to understanding the behavior of the membrane and fouling during ultrafiltration, where the chemistry of the membrane surface and the solution environment are responsible for the membrane–solute interactions.

In summary, Table 3 shows the maximum removal efficiency of various dyes and the experimental parameters used in the PEUF process.

## 6. Computational Studies

The polymer–dye interactions are one of the cornerstones of the application of the PEUF technique for the ionic dyes removal from aqueous solutions. However, a complete view about these phenomena is not possible only taking into account an experimental point of view. Therefore, the success of computational chemistry and physics software, together with the development of new methods in theoretical chemical physics, have allowed a better understanding of interactions in solutions and prediction of important properties through novelty computational analysis. In this context, the molecular docking technique is used as an attractive scaffold to understand the dye–polymer interactions as well as to estimate the lowest conformations of the system. These conformations can be evaluated over time using more robust methodologies such as molecular dynamics (MD) simulations. MD simulations enable the determination of atom and molecule trajectories, where inter-particle forces and the corresponding potential energies are computed by applying interatomic potentials or molecular mechanics force fields [111,112]. Following this protocol, Cojocaru [113] studied the binding assessment of MB dye to human serum albumin (HSA) and poly (acrylic acid) (PAA) using the complexation-ultrafiltration process. To evaluate the intermolecular forces, docking studies (using the AutoDock-VINA algorithm) [114] were used. Here, it was possible to identify the binding regions with the following sub-domains: b1 (IIA + IIB), b2 (IB + IIA), b3 (IB + IIIA), and b4 (IB), where the best pose is the b1 region. In this conformation, the MB–polymer complex is primarily stabilized by electrostatic interactions. Additionally, the hydrophobic contacts present are mediated by the contact between non-polar groups of atoms (CH_3_, CH_2_, HCR_3_, and aromatic ring carbons). The MD calculations were done with the YASARA-Structure program; these studies revealed the stability of the HSA–MB complex in the explicit solvent. Density functional theory (DFT) calculations were also performed, and gave a reliable picture of frontier orbitals (HOMO → LUMO) of the MB molecule. The HOMO → LUMO configuration was assigned to π → π* electronic transitions. In this line, this researcher also investigated the removal of anionic dye (AO7) from aqueous solutions using PEI as a chelating agent using the complexation-ultrafiltration process [82]. Herein only computer-aided molecular docking analysis was used to explore the intermolecular interactions. The docked complexes were mainly stabilized by hydrophobic interactions. A hydrogen bond (H-bond) was found to form between an oxygen atom from the sulfonate group of Acid-Orange 7 and a secondary amine of polyethyleneimine. The use of the YASARA program allowed the researchers to estimate quantitatively the contribution of intermolecular interactions, obtaining the van der Waals (VdW) and Coulomb (electrostatic) energies. From this analysis, it was found that the electrostatic interactions play the most important role during the complexation of the AO7 molecule with PEI. Similarly, alternative theoretical approaches have been used for the study of ultrafiltration processes. In this context, Dasgupta [73] used a hybrid neural-genetic algorithm based evolutionary approach to evaluate the capability of the PEUF technique to generate dye rejection of a recalcitrant, low molecular weight RR 120. A stochastic search combined with artificial neural network (ANN) was used to evaluate theoretically the retention of RR 120 and optimize this process. The better-calculated performance index (PFI) was verified experimentally. This demonstrates the feasibility of using hybrid strategy-based predictions to study the membrane performance.

## 7. Conclusions and Perspectives

It is concluded that the PEUF technique is effective in removing ionic dyes from aqueous solutions. This seems a promising technique to be examined in wastewater purification. Studies reported in the literature adequately expose the functioning of the PEUF technique and the mechanism by which dyes are removed from waters. From the literature it can be said that the interactions that are generated between the different soluble polymers and the dyes, either cationic or anionic, are essential in the process. Process parameters such as pH, ionic strength, interfering ions, initial color concentration, and MWCO of the ultrafiltration membrane are also key, which are generally the main objects of study, since they directly affect the performance and efficiency of the removal process. However, it is considered that one of the challenges in the research of the PEUF technique to remove dyes is to be able to scale up and apply it at an industrial level, since no publication so far evaluates the implementation of this technique at this level, and the results of current publications show that it is an efficient technique that can combine synthetic and natural polymers to remove dyes and other pollutants present in wastewater. Few studies have been reported on the removal of dyes in aqueous solution using the PEUF technique, which suggests that this is an area in which further research is needed to develop this technique effectively with scaling-up projections. That is why research is encouraged to continue to develop systems using only natural polymers to increase the sustainability of the technique. From a theoretical point of view, the study of the PEUF process remains in an early stage. In this context, the most important contributions are consolidated by the use of molecular docking analysis, molecular dynamics simulations, DFT calculations, and hybrid neural-genetic algorithms based on an evolutionary approach. However, there are few theoretical reports about the polymer–dye interactions when the PEUF technique is used. In the majority, the analysis of intermolecular forces present is limited from the experimental view, which in principle prevents a complete view about the involved phenomena. We encourage the scientific community to make broad use of these methodologies as well as the new approximations implemented today.

## Figures and Tables

**Table 1:** Types of anionic and cationic dyes and their molecular structures.

Dyes	Molecular Structure	References
**Anionic**		
MO	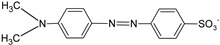	[2,21,77,78,79]
[2,21,77,79] DB71	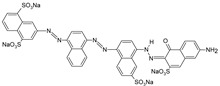	[2]
EBBR	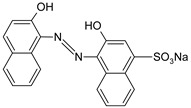	[72,80]
RR 120	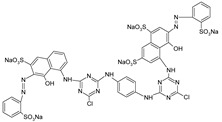	[72,80,81]
AO7	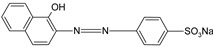	[82]
IC	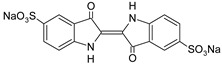	[77]
AB	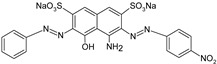	[77]
DB	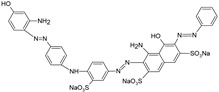	[77]
RB_5_	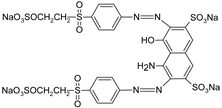	[83]
AB129	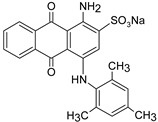	[70]
OII	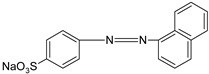	[61,84]
EO	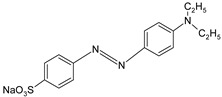	[61,84]
**Cationic**		
ST	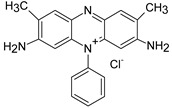	[72,80]
MG	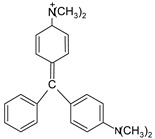	[85]
BG	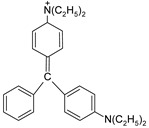	[85]
NF	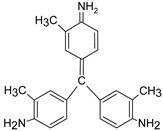	[85]
CV	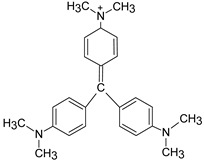	[70,86]
MB	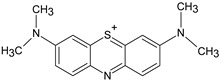	[28,74,76]
TB	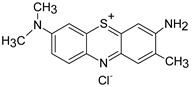	[21,33,74]
AY	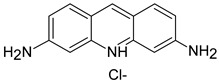	[74]

**Table 2:** Polymers and biopolymers used in the removal of dyes in PEUF.

**Anionic WSP**	**Basic Dyes**	**References**
PAA 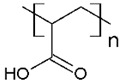	CV, ST, TB and MB	[21,31,72,80]
SA 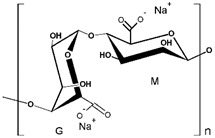	MB	[28,31]
PAMPS 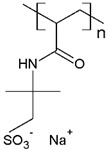	MB	[76]
PVS 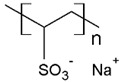	MB	[76]
PSS 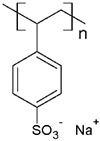	MB, NF, BG and MG	[75,85]
PANH_4_ 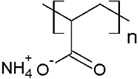	MB, ST, CV and TB	[21,31,72]
**Cationic WSP**	**Acid Dyes**	**References**
PEI 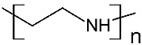	AO7 and RR 120	[6,73,82]
Poly(2-diethylaminoethyl methacrylate) (PDEAEMA) 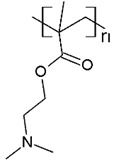	OII and EO	[61,84]
CHI 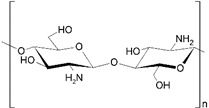	RB_5_, DB71 and MO	[2,83]

**Table 3:** Comparative table showing the removal percentage of ionic dyes reported in the literature and experimental parameters used in the PEUF process.

WSP	Dye	Membrane	pH	Initial Dye (mg L^–1^)	Polymer Dosage (mg L^−1^)	T (°C)	Time (min)	Retention Rate (%)	Ref.
CHI	MO	Cellulose	4.5	32.7	116	25	5–80	86	[2]
	DB71	10 kDa		102.9				89	
PEI	RR 120	PES-30	8.4	50	50	35	10	99	[6]
CHI		30 kDa	6.3					88	
SA	MB	Cellulose	8	50	1250	20	---	98	[28]
		10 kDa							
PAA	MB	Cellulose	4.06	31.9	28.8	25	10–100	99	[31]
PANH_4_		10 kDa	8.24		35.6			98	
PAA	TB	---	2–6	27	8.6	25	180	89	[33]
			4.5						
	MO		2–6	32.7	14.4			86	
			4.5						
PEG	MO	Cellulose	4	163.6	3360	---	30	65	[78]
		10kDa							
PANH_4_	ST	Cellulose	4–10	20	10	---	10–90	99	[80]
	EBBR	10kDa	6–10	10	30	---	Oct-90	90	
PEI	RR120	PES-30	10	50	150	35	10	99.94	[81]
		30 kDa							
PAA	CV	Oss-flow	10–12	2	20	---	30	95	[86]
		10kDa							
